# Antioxidant Activity and Main Chemical Components of a Novel Fermented Tea

**DOI:** 10.3390/molecules24162917

**Published:** 2019-08-12

**Authors:** Tao Tong, Ya-Juan Liu, Jinhong Kang, Cheng-Mei Zhang, Seong-Gook Kang

**Affiliations:** 1 Beijing Advanced Innovation Center for Food Nutrition and Human Health, College of Food Science and Nutritional Engineering, China Agricultural University, Beijing 100083, China; 2 Department of Food Engineering, Mokpo National University, 61 Dorimri, Chungkyemyon, Muangun, Jeonnam 534-729, Korea; 3 College of Pharmacy, Korea University, Sejong 30019, Korea,

**Keywords:** *Camelia sinensis*, fermented tea, antioxidant, catechin, theaflavin, γ-aminobutyric acid

## Abstract

In the present study, we aimed to develop a novel fermented tea (NFT) product and to evaluate their in vitro antioxidant potential and chemical composition. We found that NFT contained a high level of total phenolic compounds (102.98 mg gallic acid equivalents/g extract) and exhibited diverse antioxidant activities, such as scavenging of 1,1-diphenyl-2-picryl-hydrazyl (DPPH) and hydroxyl radicals, as well as reducing power. The total catechins in NFT were comparable to those of Lipton black tea (LBT), but lower than those of Boseong green tea (BGT) or Tieguanyin oolong tea (TOT). Among all catechins tested, epigallocatechin (EGC) and epigallocatechin-3-O-gallate (EGCG) were the predominant compounds in NFT. In particular, the contents of total theaflavins (TFs), theaflavin (TF), theaflavin-3-gallate (TF3G), and theaflavin-3’-gallate (TF3’G) in NFT were significantly higher than that of BGT, TOT, or LBT. NFT had the highest level of total essential amino acid and γ-aminobutyric acid (GABA) compared with BGT, TOT and LBT. Furthermore, the sensory evaluation results showed that NFT had satisfactory color, aroma, taste, and overall acceptability scores. Our results highlight the potential usefulness of this novel fermented tea as a nutraceutical food/ingredient with special functional activities.

## 1. Introduction

Tea is produced from the plant *Camelia sinensis* and is one of the most popular beverages worldwide, second only to water [1]. It contains as much as 30% soluble ingredients, which may vary with the cultivar, climate condition, genetic strain, production region, plucking season, position of the leaf as well as the processing operations [2]. Polyphenols, the main constituents of tea, is known to take up 20–35% of tea’s dry weight. Among polyphenols, catechin was the predominant group, counting for 60–80% of tea polyphenols. In general, catechins largely include catechin, epicatechin (EC), epicatechin gallate (ECG), epigallocatechin (EGC), and epigallocatechin-3-O-gallate (EGCG) [2,3,4]. Tea is typically categorized into seven types based on processing methods: green tea, yellow tea, white tea, oolong tea, black tea, aged pu-erh tea, and ripened pu-erh tea [5]. During the processing of tea, fermentation changes the chemical composition of tea, leading to the production of theaflavins [6]. 

Tea polyphenols have attracted huge interest because of their presumed associated health properties [7,8]. Over the past few years, numerous studies have indicated that catechins and other polyphenols in tea exhibit powerful antioxidant activities [9]. They function as antioxidants in vitro by scavenging nitrogen species and reactive oxygen generated due to a variety of oxidative stress and by sequestering metal ions [10]. They may also act indirectly as antioxidants by their impacts on transcription factors and enzyme activities [11]. Therefore, regular consumption of tea is viewed as an option to improve antioxidant status in vivo and to lower the risk of certain types of cancer and coronary heart disease [12]. 

In the present study, we aimed to develop a novel fermented tea (NFT) product based on tea leaves (*Camellia sinensis* L.) from Boseong-gun, Jeonnam, South Korea. The in vitro antioxidant potential, total phenolic and flavonoid contents, amino acid composition, and sensory characteristics of NFT were evaluated and were compared with Boseong green tea (BGT), Tieguanyin oolong tea (TOT) and Lipton black tea (LBT). For comparison, Boseong green tea was selected as the common green tea preferred by Koreans. And Tieguanyin oolong tea was selected to represent popular oolong tea. Lipton black tea was selected as one of the well-known black tea. These teas are commonly sold in shops for personal consumption.

## 2. Results and Discussion 

### 2.1. Proximate Composition of Tea Leaves and Soluble Solid Contents of Tea Infusions

As stated in Table 1, there was significant difference among the tea samples in terms of the moisture content: NFT had the lowest moisture content, compared to BGT, TOT, or LBT. The ash content of NFT was higher than that in TOT or LBT, but lower than that in BGT. There was no significant difference among the tea samples in fat contents (Table 1). The crude protein content in NFT was significantly higher than that in the other tea samples. The carbohydrate content in NFT was comparable to that of BGT or TOT, but lower than that of LBT (Table 1). On the other hand, we found that the soluble solid content in NFT infusions was similar to that in BGT, TOT, or LBT infusions (Table 1).

### 2.2. DPPH Radical Scavenging Capacity

**Figure 1:**
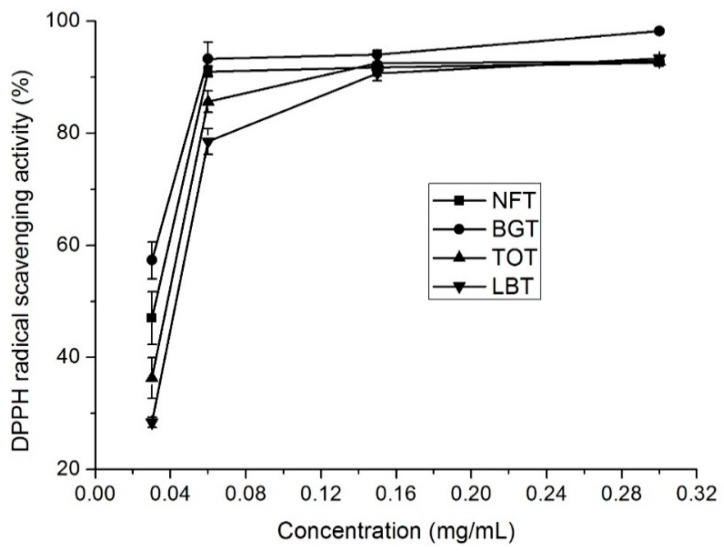
DPPH radical scavenging activity of different tea samples. NFT, novel fermented tea; BGT, Boseong green tea; TOT, Tieguanyin oolong tea; LBT, Lipton black tea.

The stable organic free radical, DPPH, has been considered as a useful reagent for determining free radical scavenging capacity of antioxidant materials. In general, the scavenging capacities of NFT, BGT, TOT, and LBT on DPPH radicals were evident at all tested concentrations (Figure 1). All the tea infusions showed >70% radical scavenging capacities at 0.06 mg/mL concentration. DPPH radical scavenging capacity showed a concentration dependency and increased with increase in concentration. The DPPH scavenging capacity of NFT was medium (46%) at 0.03 mg/mL and, at a concentration of 0.06 mg/mL, reached a plateau of 91% (Figure 1). EC_50_ values (the effective concentration of 50% inhibition) for NFT, BGT, TOT, and LBT infusions were 0.045, 0.029, 0.062, and 0.077 mg/ mL, respectively (Table 2). 

### 2.3. Reducing Power

**Figure 2:**
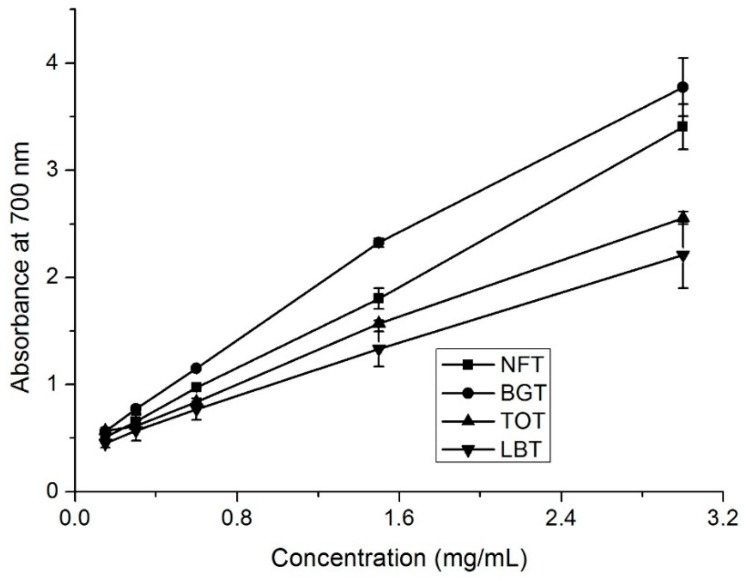
Reducing power of different tea samples. NFT, novel fermented tea; BGT, Boseong green tea; TOT, Tieguanyin oolong tea; LBT, Lipton black tea.

In the reducing power assay, the presence of antioxidants in the extracts results in the reduction of the Fe^3+^/ferricyanide complex to its ferrous form [13]. Figure 2 presented the extent of the reduction, in terms of absorbance values at 700 nm, for the tea infusions ranging at concentration from 0.5 to 3 mg/mL. The reducing power of the samples correlated well with increasing concentrations (Figure 2). BGT showed the strongest reducing power, followed by NFT (Figure 2). EC_50_ values (the effective concentration at which the absorbance value was 0.5) of the reducing power values for NFT, BGT, TOT, and LBT infusions were 0.125, 0.072, 0.127, and 0.194 mg/ mL (Table 2), respectively.

### 2.4. Hydroxyl Radical Scavenging Activity

**Figure 3:**
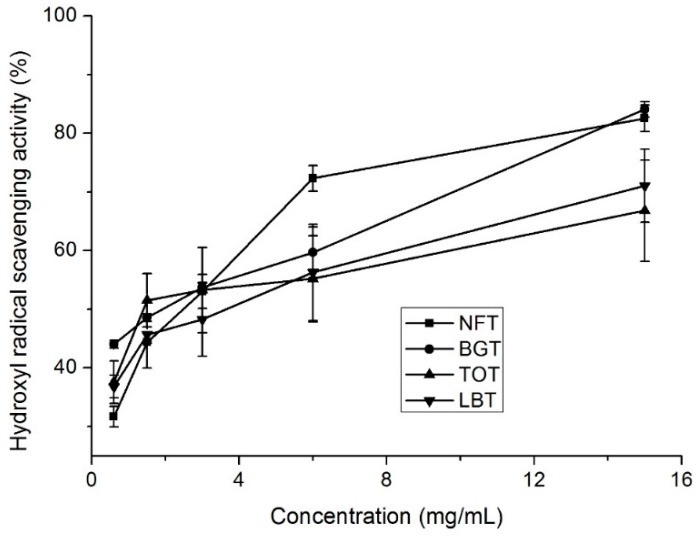
Hydroxyl radical scavenging activity of different tea samples. NFT, novel fermented tea; BGT, Boseong green tea; TOT, Tieguanyin oolong tea; LBT, Lipton black tea.

The hydroxyl radical, known to be generated through the Fenton reaction in this system, was scavenged by tea infusions. The scavenging effect of all samples was shown in Figure 3. For all the samples, the effects of scavenging hydroxyl radicals were in a concentration-dependent manner. EC_50_ values (the effective concentration of 50% inhibition) for NFT, BGT, TOT, and LBT infusions were 2.023, 1.39, 3.438, and 4.524 mg/mL (Table 2), respectively. 

### 2.5. Total Phenolic (TPC) and Flavonoid Contents (TFC)

Significant differences in total phenolics were noticed between the different tea infusions (Table 3). Briefly, the TPC of BGT were highest (128.44 mg of gallic acid equivalent (GAE)/g), followed by NFT (102.98 mg of GAE/g) and LBT (83.71 mg of GAE/g). TOT had the lowest TPC (75.63 mg of GAE/g). TFC in tea infusions ranged from 14.92 to 30.31 mg of rutin equivalents (RE)/g. TFC in tea infusions were not in proportion to TPC: BGT had the highest TFC (30.31 mg of RE/g), followed by TOT, LBT, and NFT (Table 3).

### 2.6. Catechins, Caffeine, and Theaflavins Contents

NFT, together with BGT, TOT, and LBT, contained different total phenolic and total flavonoid contents and exhibited favorable antioxidant capacities (Table 3 and Figure 1, Figure 2 and Figure 3). Next, HPLC analysis of tea samples were conducted to identify and compare major phenolic compounds including catechins, caffeine, and theaflavins. 

Catechins (EGC, C, EGCG, EC and ECG) have been regarded as the major phenols in almost all kinds of teas. The contents of five major catechins in tea infusions were determined and the results were shown in Table 4. Among all catechins tested, EGC and EGCG were the two predominant types of catechin in NFT, BGT, TOT, and LBT infusions accounting for 62, 78, 85, and 70% of the total catechins, respectively (Table 4). Similarly, Koch and co-workers demonstrated that EGC and EGCG were the two major catechins in several green and black tea samples from different cultivation areas, although they found that the primary catechin in both green and black tea investigated is not EGCG but EGC [14,15]. EGC and EGCG have also been reported to be the major catechins in the final Fuzhuan brick teas samples [16]. The level of EGC in NFT (1013 mg/100 g) was higher than that in LBT (817 mg/100 g), but lower than that in BGT (4020 mg/100 g) and TOT (2604 mg/100 g). The EGCG content of NFT was significantly lower compared to that of BGT, TOT, or LBT. The level of C and ECG in NFT was lower than that of BGT, but higher than that of TOT and LBT (Table 4). EC ranged from 248 mg/100 g in NFT to 1086 mg/100 g in BGT. The contents of total catechins in NFT was comparable to that of LBT, but lower than that of BGT or TOT. The contents of caffeine are at the same level in NFT and BGT (Table 4). Previous study demonstrated that the concentration of major phenolic compounds including EC, ECG, EGC, and EGCG in black tea infusion is related to the brewing time and that after 2 min brewing, most phenolics had already been extracted, and extract composition did not significantly change at a prolonged extraction time (4 min) [17]. Future study aiming to determine the effect of brewing time on the composition and antioxidant properties of NBT is warranted.

During the fermentation process, catechins were degraded into the B ring fission catechins derivatives and polymerize into theaflavin derivatives by polyphenol oxidase or peroxidase. Theaflavin levels are known to be directly related to the taste and quality of the tea [3]. Therefore, we decided to determine the TFs contents in the tea infusions. We found that total TFs and TF are present in very low concentration in BGT and TOT infusions. In contrast, NFT had the highest contents of total TFs, TF, TF3G, and TF3’G, followed by LBT (Table 4). Liu et al. reported that with increasing semi-fermentation time, the total catechin concentration decreased, while total theaflavins contents increased significantly [18].

Although functional studies on TFs have lagged seriously behind those of the catechins, TFs have attracted considerable interest recently, as they are reported to play important physiological roles, such as antioxidant [19], anticancer [20], anti-atherosclerotic [21] activities as well as the prevention of osteoporosis [22]. Furthermore, these compounds have human health benefits including glucose-lowering [23] and anti-obesity effects [24], and are useful in the prevention of lifestyle-related diseases. Takemoto et al. revealed that, the extraction of TFs from black tea leaves at sufficient levels for use in medical studies has been difficult due to the low concentration of TFs present in black tea [25]. In the present study, we provided a novel tea product containing TFs in high concentration (Table 4), encouraging future studies to extract theaflavins from NFT for pharmaceutical use or to develop new theaflavin-enriched functional foods.

The composition of bioactive substances in tea infusions is thought to contribute to its antioxidant activities. While numerous reports have indicated that phenolic compounds presented in the tea such as catechins and TFs are the contributor to the antioxidant capacities of tea, it should be noted that the presence of non-phenolic components such as polysaccharides may also contribute to the antioxidant activity. Sun et al. reported that four kinds of green tea polysaccharides, with Mw of 10.88, 8.16, 4.82, and 2.31 kDa, respectively, possessed hydroxyl and ABTS radical scavenging activity and reducing power [26].

### 2.7. Free Amino Acids Analysis

Amino acids are important bioactive components of tea and are known to play an important role in the taste of tea. Here, significant differences in total free amino acids were observed among the four kinds of tea samples. NFT had the highest content of total free amino acids (630 mg/100 g), followed by BGT (598 mg/100 g), LBT (150 mg/100 g), and TOT (70 mg/100 g) (Table 5). According to the chemical structure and biological activity, different amino acids commonly are divided to two groups: essential amino acids and non-essential amino acids. Among essential amino acids, NFT had the highest level of Val, Thr, Ile, Leu, Phe, Lys, and Met compared to BGT, TOT, or LBT. Met was not detected in TOT and LBT but existed in NFT and BGT in a low concentration (Table 5). 

GABA is a non-proteinaceous amino acid that occurs in animals, plants, and bacteria [27]. It is primarily produced by microorganism and the biosynthesis of GABA is one step reaction of decarboxylating glutamate to GABA, catalyzed by glutamate decarboxylase. GABA generally occurs at a very low level in plants but its content increases substantially after exposure to a range of stresses, especially oxygen-deficiency [27]. In the present study, NFT had the most abundant GABA (113.8 mg/100 g), followed by BGT (8.59 mg/100 g), LBT (5.82 mg/100 g), and TOT (1.97 mg/100 g) (Table 5). Compared with previous studies on other types of tea, the level of GABA in fermented tea was much higher than that of pu-erh tea and white tea [28].

GABA is well known to function as inhibitory neurotransmitters in the central nervous system [29] and play multiple positive roles, such as reducing hypertension, regulating blood pressure, and ameliorating autism [30,31,32]. Recently, the development of functional foods containing GABA in high concentration has gained popularity in complementary medicine practices as an accessible intervention to reduce the impact of chronic stress-induced autonomic imbalance and increased risk for cardiovascular disease. Several commercially available GABA-enriched foods have been developed such as GABA tea [33], rice germ [34], tempeh-like fermented soybean [35], and black raspberry juice [36]. Here, we demonstrated that NFT contains high level of GABA (Table 5), highlighting the potential usefulness of NFT for the prevention or treatment of chronic stress-induced autonomic disorders and increased risk for cardiovascular disease. 

### 2.8. Color Difference Analysis of Tea Infusions and Sensory Quality

Lightness (L*) is the measurement of the white-black color, so that a decrease on the L* values indicates darkening. As can be observed in Table 6, the L* value of fermented tea infusions was slightly higher than that of LBT infusions, but lower than those of BGT and TOT infusions. Redness (a*) is a parameter that describe the color of samples in the red-green axis. The a* value of NFT infusions was slightly higher than those of BGT and TOT infusions, but lower than that of LBT infusions (Table 6). Yellowness (b*) is a parameter that measures color changes in the yellow-blue range, becoming more yellowish as the numbers increase. The TOT infusions had the highest b* value, followed by NFT, LBT, and BGT infusions (Table 6).

It has been established that sensory characteristics are known to be correlated with chemical constituents, which are mostly polyphenols, including catechins, theaflavins, and thearubigins, as well as free amino acids, sugars, organic acids, and caffeine [37]. In the present study, the NFT had satisfactory color, aroma, taste, and overall acceptability scores (Table 7). In addition, we found that BGT had highest bitterness and astringency scores compared to NFT, TOT, and LBT (Table 7). This is consistent with the aforementioned results that the green tea had the highest catechins contents. Tea polyphenols, particularly tea catechins, have been found to activate the human bitter taste receptors hTAS2R14 [38] and to be responsible for bitterness and astringency [39,40]. 

## 3. Materials and Methods 

### 3.1. Reagents

Aluminum nitrate nonahydrate, aluminium chloride, 2,2-diphenyl-1-picryl-hydrazyl (DPPH), ferric chloride, ferrous sulfate, gallic acid, Folin–Ciocalteu’s reagent, 3,5-dinitrosalicylic acid, hydrogen peroxide, potassium dihydrogen phosphate, potassium ferricyanide, sodium salicylate, sodium carbonate, sodium nitrite, trichloroacetic acid, amino acids [alanine (Ala), aspartic acid (Asp), arginine (Arg), histidine (His), gamma-butyric acid (GABA), lysine (Lys), glutamic acid (Glu), serine (Ser), threonine (Thr), theanine (Thea), tyrosine (Tyr)], and caffeine were obtained from Sigma Chemical Co. (St. Louis, MO, USA). Catechins [(+)-catechin (C), (-)-epicatechin (EC), (-)-epigallocatechin (EGC), (-)-epicatechingallate (ECG), and (-)-epigallocatechin gallate (EGCG)] and theaflavins [theaflavin (TF), theaflavin-3-gallate (TF3G), and theaflavin-3’-gallate (TF3’G)] were obtained from Wako Pure Chemical Industries, Ltd. (Osaka, Japan). All other chemicals and solvents used were of standard analytical grade.

### 3.2. Samples and Preparation of Tea Infusions

**Figure 4:**
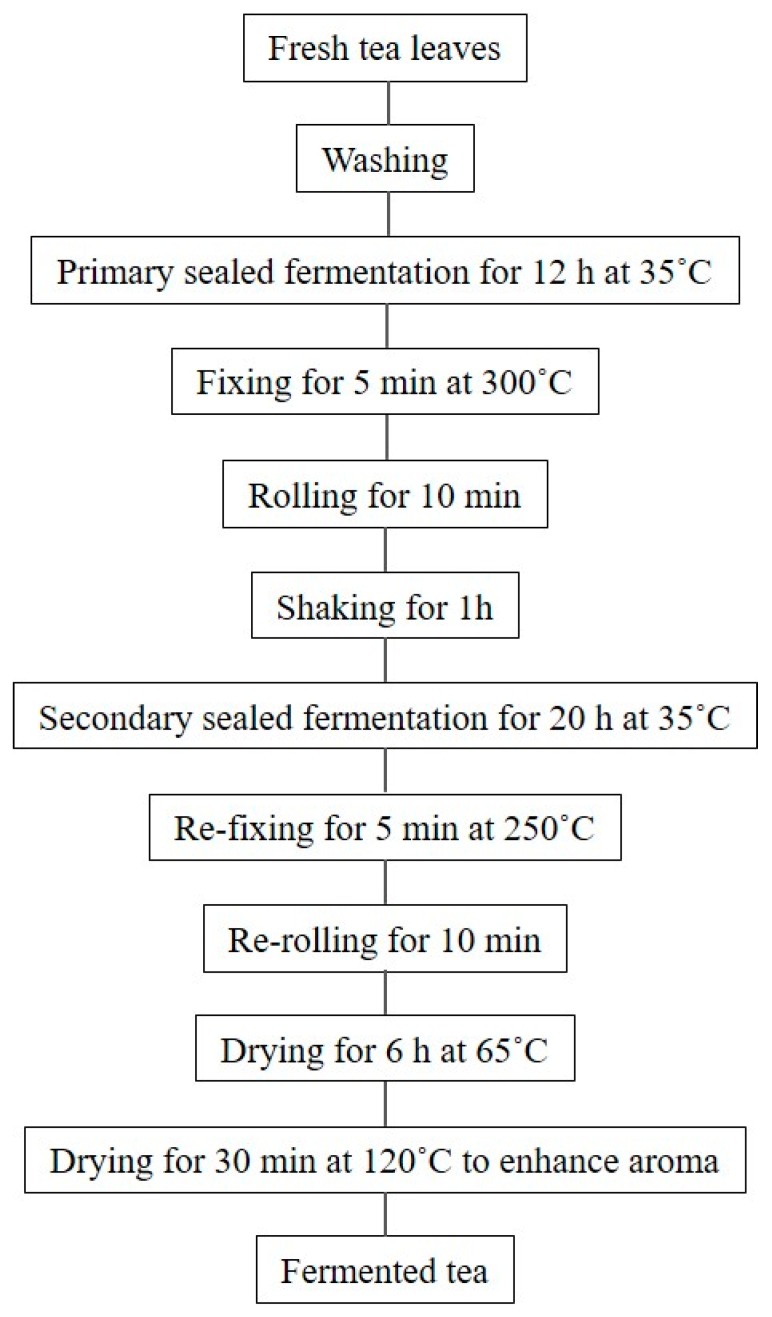
Flowchart of NFT preparation from fresh tea leaves.

This study was conducted during tea plucking season. Young tender tea leaves (*Camellia sinensis* L.) from tea plants in Boseong-gun, Jeonnam, South Korea were plucked in April, 2015. The fermented tea was prepared according to processing chart (Figure 4). The processing method used in the present study was initially designed by GreenteaWorld Co. (Boseong-gun, Jeonnam, South Korea) and optimized by us through preliminary test. Boseong green tea, Tieguanyin oolong tea, and Lipton black tea were purchased from the local market and used as comparison. For the infusion, 1.5 g of tea leaves were dunked into 50 mL of boiling deionized water. The mixtures were kept boiling for 20 min on a hot plate and filtered through Whatman No. 2 filter paper to remove tea leaves. The tea infusions were used for evaluation of antioxidant activities, chemical composition contents, color, sensory characteristics.

### 3.3. Analysis of Proximate Composition and Soluble Solid Content

The moisture, crude fat, protein and ash contents of tea leaves were analyzed in accordance with the standard method of the AOAC [41]. Carbohydrate content was obtained by difference. The soluble solid content (°Brix) was determined by a refractometer (AR200 Reichert, USA).

### 3.4. Determination of DPPH Radical Scavenging Activity

The DPPH radical scavenging activity was determined by the method of Tong et al. [42]. One milliliter of reaction mixture contained 0.5 mL of 0.1 mM DPPH solution and 0.5 mL tea infusions. The mixtures were shaken vigorously and placed in darkness. Absorbance was measured at 517 nm after 30 min and the inhibition ability was obtained from the formula (1):

% inhibition = [(Abs_0_−Abs_1_)/Abs_0_] × 100 (1)

Abs_0_: absorbance without samples; Abs_1_: absorbance in the presence of the samples

### 3.5. Determination of Hydroxyl Radical Scavenging Activity

Hydroxyl radical scavenging activity was assayed by the method of Smirnoff and Cumbes [43]. The reaction mixture contained 500 μL of 1.5 mM ferrous sulfate, 150 μL of 20 mM sodium salicylate, 500 μL tea infusions, and 350 μL of 6 mM hydrogen peroxide. After incubation for 1 h at 37 °C in a water bath, the absorbance of the reaction mixture was measured at 562 nm using a spectrophotometer (Model 8453, Agilent Technologies, Inc., Palo Alto, CA, USA) the inhibition ability was obtained using formula (1).

### 3.6. Measurement of Reducing Power

The reducing power of tea infusions were determined by the previously described method [44]. The tea infusions (500 μL) were placed in a tube, to which 1.25 mL of phosphate buffer solution (0.2 M, pH 6.6), as well as 1.25 mL of 1% potassium ferricynide solution were added. After incubation at 50 °C for 20 min, 1.25 mL of trichloroacetic acid solution were added to the tube. 1.25 mL of supernatant obtained by centrifugation at 3000 rpm for 10 min was diluted with 1.25 mL of deionized water. Finally, 0.25 mL of 0.1% ferric chloride solution was added to complete the assay. The absorbance was determined at 700 nm and represented the reducing power. 

### 3.7. Determination of Total Phenolic Content (TPC)

The total phenolic content of each tea infusion was determined by Folin–Ciocalteu method as described by Singleton and Rossi [45]. The tea infusion (100 μL) was mixed with 7.9 mL of distilled water and 0.2 mL of Folin–Ciocalteu’s reagent. After 5 min, 1 mL of 15% NaCO_3_ was added and the mixture was incubated for 2 h in darkness at room temperature. The absorbance of mixture was measured at 765 nm. The concentration of TPC was calculated as mg of gallic acid equivalent by using an equation obtained from gallic acid calibration curve (y = 33.887x + 0.0431, R^2^ = 0.9986).

### 3.8. Determination of Total Flavonoid Content (TFC)

The total flavonoids content of each tea infusion was estimated by method described by Jia et al. [46]. One mL of tea infusion was mixed with 4 mL of distilled water and 0.3 mL of 5% NaNO_2_ solution. After 5 min, 0.3 mL of 10% AlCl_3_ solution was added, and the mixture was allowed to stand for 6 min before the addition of 2 mL of 1 M NaOH. The total volume of mixture was made up to 10 mL with distilled water, and then the absorbance was measured at 510 nm using a UV-visible spectrophotometer (U-1100, Hitachi, Japan). Rutin was used as standard for a calibration curve (y = 1.1962x + 0.0017, R^2^ = 0.9998). 

### 3.9. Determination of Catechins, Caffeine, and Theaflavins Contents

The catechin, caffeine, and theaflavin contents in tea samples were quantitatively analyzed by octadecylsilane-high performance liquid chromatography (ODS-HPLC). The tea leaves (3 g) was extracted with hot distilled water (100 mL) for 20 min at 100 °C and filtered through membrane filter. The residue was re-extracted with hot distilled water (100 mL) by the same extraction procedure as above. The tea extracted solution were combined and its portion (1 mL) was filtered through a Millipore membrane (0.45 μm; Berrica, MA, USA). The filtrate was subjected to the ODS-HPLC (SPD-M20D; Shimadzu, Kyoto, Japan). The compounds were separated on a Shim-pack Prep-ODS(H)∙kit (4.6 mm i.d. × 250 mm, 5 μm; Shimadzu). The elution was accompanied with a gradient system of 100% H_2_O containing 20 mM KH_2_PO_4_ (eluent A) to 100% MeCN (eluent B) as follows: Started at A/B = 97:3 (*v*/*v*) for 10 min and increased to A/B = 75:25 (*v*/*v*) for 90 min. The compounds were monitored at 280 nm and the flow rate was 1.0 mL/min. The calibration curves were constructed using external standards of catechins, caffeine, and theaflavins (0.1–10 μg). The contents of catechins, caffeine, and theaflavins in tea samples were determined through triplicate experiments.

### 3.10. Analysis of Amino Acid

Free amino acid components were determined on an amino acid analyzer (Hitachi L-8900, Tokyo, Japan). Five milliliters of tea infusions were evaporated, and the dried samples were dissolved in 0.02 N HCl solution. The samples were filtered through a 0.22 μm Millipore filter before injection. The relevant configurations were a single microbore stainless-steel column (2.6 mm inside diameter, 15 cm length) with a maximum of five programmable eluting buffers and one regenerant. The column material was a Hitachi custom cation-exchange #2619F resin. Buffers and ninhydrin flow rates were 0.25 and 0.30 mL/min, respectively. In brief, amino acids separated by cation-exchange chromatography were detected spectrophotometrically after postcolumn reaction with ninhydrin reagent.

### 3.11. Analysis of the Color Difference of Tea Infusions and Sensory Evaluation

The color parameters were measured with a colorimeter (Konica-Minolta, CM3500d, Minolta, NJ, USA). The color was quantified according to the Hunter color values L∗, a∗, and b∗, where L∗ is the change in lightness from black to white; a∗ and b∗ indicate red (+a∗), green (−a∗), yellow (+b∗), and blue (−b∗) [47]. The samples were scanned six times to obtain the mean Hunter L∗, a∗, and b∗ values, and distilled water was used as reference. The sensory evaluation was carried out on the tea infusion samples within 1 h of brewing. Samples were evaluated by the students from Department of Food Engineering at Mokpo National University. Sensory attributes of the tea infusion, including color, taste, aroma and overall preference were measured using a 5-point hedonic scale with 1, 3 and 5 representing extremely dislike, neither like not dislike and extremely like, respectively.

### 3.12. Statistical Analysis

Data are expressed as mean ± standard deviation. Differences between groups were determined using one-way ANOVA (Duncan’s Multiple Range Test). The p values < 0.05 were considered to be statistically significant. Data were analyzed using IBM SPSS (version 21.0 for windows, SPSS Inc., CO, USA). EC_50_ values were obtained by linear regression analysis using Origin Pro 8.5 (Origin Lab Corporation, MA, Northampton, USA).

## 4. Conclusions

To conclude, the present study comparatively evaluated the in vitro antioxidant capacity, phenolic profile, amino acid composition as well as the sensory characteristics of NFT relative to BGT, TOT, and LBT. HPLC analysis showed that among catechins tested, EGC and EGCG were the predominant compounds in NFT. The level of total catechins in NFT was significantly higher compared to LBT, but lower than that of BGT or TOT. Compared with BGT, TOT, or LBT, NFT was found to contain the most abundant total TFs, TF, TF3G, TF3’G, total essential amino acid, and GABA. Moreover, the sensory evaluation results showed that NFT had satisfactory color, aroma, taste, and overall acceptability scores. The present study developed a new fermented tea product, which has potential for protecting living bodies against oxidative damage as well as a range of oxidative damage-induced diseases. Additional research is required to further determine the potential for developing NFT as a nutraceutical or pharmaceutical ingredients.

## Figures and Tables

**Table 1:** Proximate composition of tea leaves and soluble solids contents of tea infusions.

Parameter	NFT	BGT	TOT	LBT
Moisture, %	2.38 ± 0.1^d^	2.79 ± 0.01^c^	6.25 ± 0^b^	6.41 ± 0.02^a^
Ash, %	5.17 ± 0.06^b^	5.4 ± 0.01^a^	3.99 ± 0.03^d^	4.91 ± 0.15^c^
Fat, %	0.72 ± 0.18^a^	2.12 ± 1.36^a^	5.91 ± 3.89^a^	1.79 ± 0.79^a^
Protein, %	35.78 ± 0.83^a^	33.55 ± 0.59^b^	21.37 ± 0.13^c^	19.45 ± 0.55^d^
Carbohydrate, %	55.94 ± 0.84^b^	56.14 ± 1.92^b^	62.49 ± 3.99^ab^	67.45 ± 1.51^a^
Soluble solids, °Brix	1.35 ± 0.21^ab^	1.6 ± 0.14^a^	1 ± 0.14^b^	1.4 ± 0.14^ab^

Values are the means ± standard deviation of triplicate determinations. Means with the different letters (a, b, c, or d) in the same row are significantly different at p < 0.05. NFT, novel fermented tea; BGT, Boseong green tea; TOT, Tieguanyin oolong tea; LBT, Lipton black tea.

**Table 2:** EC_50_ values of DPPH, hydroxyl radical scavenging activities, and reducing power.

Antioxidant Activity	NFT	BGT	TOT	LBT
DPPH radical scavenging activity	0.045 ± 0.01^c^	0.029 ± 0.01^d^	0.062 ± 0.01^b^	0.077 ± 0.01^a^
Reducing power	0.125 ± 0.02^b^	0.072 ± 0.03^a^	0.127 ± 0.02^b^	0.194 ± 0.08^c^
Hydroxyl radical scavenging activity	2.023 ± 0.17^c^	1.39 ± 0.96^d^	3.438 ± 3.25^b^	4.524 ± 2.88^a^

Values are the means ± standard deviation of triplicate determinations. Means with the different letters (a, b, c, or d) in the same row are significantly different at p < 0.05. NFT, novel fermented tea; BGT, Boseong green tea; TOT, Tieguanyin oolong tea; LBT, Lipton black tea.

**Table 3:** Total phenolic and flavonoid contents of different tea samples.

Parameter	NFT	BGT	TOT	LBT
TPC (mg of gallic acid equivalent/g)	102.98 ± 1.41^b^	128.44 ± 1.71^a^	75.63 ± 1.57^d^	83.71 ± 1.10^c^
TFC (mg of rutin equivalent/g)	14.92 ± 0.69^d^	30.31 ± 0.46^a^	28.32 ± 0.75^b^	24.80 ± 60^c^

Values are the means ± standard deviation of triplicate determinations. Means with the different letters (a, b, c, or d) in the same row are significantly different at p < 0.05. NFT, novel fermented tea; BGT, Boseong green tea; TOT, Tieguanyin oolong tea; LBT, Lipton black tea.

**Table 4:** Catechins, caffeine, and theaflavins contents in different tea samples (mg/100 g).

Parameter	Compound	NFT	BGT	TOT	LBT
Catechins	EGC	1013.08 ± 78.72^c^	4020.83 ± 103.58^a^	2604.98 ± 182.8^b^	817.36 ± 188.45^c^
	C	300.03 ± 30.55^b^	457.83 ± 14.17^a^	126.46 ± 29.66^c^	144.3 ± 56.67^c^
	EC	248.2 ± 179.29^b^	1086.33 ± 18.6^a^	463.1 ± 75.64^b^	362.86 ± 34.37^b^
	EGCG	937.93 ± 48.44^d^	6855.42 ± 43.68^a^	3046.71 ± 290.3^b^	1436.31 ± 265.85^c^
	ECG	645.8 ± 44.06^a^	1566.01 ± 6.36^b^	414.34 ± 22.14^c^	456.86 ± 36.16^c^
	Total	3145.04 ± 303.48^c^	13986.41 ± 163.16^a^	6655.6 ± 597.22^b^	3217.68 ± 326.25^c^
Caffeine		2451.66 ± 97.93^a^	2432.6 ± 16.56^ab^	1993.53 ± 22.68^c^	2206.99 ± 174.08^bc^
Theaflavins	TF	74.06 ± 6.9^a^	4.07 ± 0.02^c^	6.88 ± 0.27^c^	51.28 ± 3.45^b^
	TF3G	1008.61 ± 103.17^a^	nd	nd	404.21 ± 15.1^b^
	TF3’G	27.11 ± 4.25^a^	nd	nd	5.23 ± 2.06^b^
	Total	1109.78 ± 113.93^a^	4.07 ± 0.02^c^	6.88 ± 0.27^c^	460.73 ± 16.5^b^

Values are the means ± standard deviation of triplicate determinations. Means with the different letters (a, b, c, or d) in the same row are significantly different at p < 0.05 in a row. nd, not detected. NFT, novel fermented tea; BGT, Boseong green tea; TOT, Tieguanyin oolong tea; LBT, Lipton black tea; C, catechin; EC, epicatechin; ECG, epicatechin gallate; EGC, epigallocatechin, EGCG, epigallocatechin-3-O-gallate; TF, theaflavin, TF3G, theaflavin-3-gallate; TF3’G, theaflavin-3’-gallate.

**Table 5:** Quantification of amino acid contents in different tea samples (mg/100 g).

Parameter		NFT	BGT	TOT	LBT
Essential amino acid	Val	34.93 ± 1.99^a^	18.25 ± 0.13^b^	9.1 ± 0.52^d^	13.41 ± 0.76^c^
	Thr	18.56 ± 0.58^a^	10.08 ± 0.16^b^	3.37 ± 0.13^c^	4.3 ± 0.38^c^
	Ile	14.84 ± 0.91^a^	1.78 ± 0.11^d^	3.24 ± 0.07^c^	5.58 ± 0.49^b^
	Leu	27.9 ± 1.87^a^	2.69 ± 0.4^b^	1.76 ± 0.02^c^	2.88 ± 0.01^b^
	Phe	17.2 ± 0.8^a^	5.51 ± 0.16^c^	4.19 ± 0.28^c^	7.19 ± 1.03^b^
	Lys	31.61 ± 1.56^a^	6.59 ± 0.08^b^	2.5 ± 0.1^c^	2.55 ± 0.22^c^
	Met	2.16 ± 0.21^a^	0.24 ± 0.01^b^	Nd	Nd
	Total	147.17 ± 7.92^a^	45.12 ± 0.46^b^	24.14 ± 0.95^c^	35.9 ± 2.89b^c^
Non-essential amino acid	Asp	10.45 ± 0.32^a^	6.48 ± 0.03^b^	4.52 ± 0.2^b^	5.18 ± 0.36^b^
	Ser	31.19 ± 0.65^a^	39.37 ± 0.23^a^	7.47 ± 0.36^c^	11.67 ± 0.78^b^
	Asn	15.26 ± 0.18^a^	6.82 ± 0.16^b^	4.07 ± 0.13^c^	6.72 ± 0.83^b^
	Glu	12.89 ± 0.64^c^	89.73 ± 0.52^a^	18.61 ± 0.78^b^	21.81 ± 1.48^b^
	Gln	68.96 ± 0.94^b^	204.83 ± 8.68^a^	Nd	35.13 ± 2.67^c^
	Gly	11.86 ± 0.41^a^	2.34 ± 0.04^b^	0.63 ± 0.04^c^	0.66 ± 0.06^c^
	Ala	56.48 ± 0.91^a^	10.6 ± 0.04^b^	4.1 ± 0.13^c^	12.74 ± 0.86^b^
	Tyr	30.61 ± 1.94^a^	4.29 ± 0.9^c^	1.08 ± 0.01^d^	7.49 ± 0.19^b^
	His	5.28 ± 0.3^a^	3.82 ± 0.08^b^	Nd	Nd
	Arg	103.99 ± 6.82^b^	173.21 ± 15.67^a^	1.83 ± 0.1^d^	4.63 ± 0.45^c^
	Pro	22.24 ± 2.23^a^	3.59 ± 0.39^b^	1.95 ± 0.48^c^	2.35 ± 0.83^b^
	Total	369.19 ± 15.33^b^	545.06 ± 6.78^a^	44.25 ± 2.04^d^	108.36 ± 6.87^c^
GABA		113.8 ± 3.66^a^	8.59 ± 0.13^b^	1.97 ± 0.02^d^	5.82 ± 0.66b^c^
Total amino acids		630.16 ± 26.91^a^	598.76 ± 7.11^a^	70.35 ± 2.97^c^	150.07 ± 10.42^b^

Values are the means ± standard deviation of triplicate determinations. Means with the different letters (a, b, c, or d) in the same row are significantly different at p < 0.05. NFT, novel fermented tea; BGT, Boseong green tea; TOT, Tieguanyin oolong tea; LBT, Lipton black tea; GABA.

**Table 6:** Color values of different tea infusions.

Parameter	NFT	BGT	TOT	LBT
L	83.01 ± 0.48^c^	103.33 ± 0.44^a^	98.67 ± 0.39^b^	78.95 ± 0.65^d^
a	6.9 ± 0.03^b^	–2.12 ± 0.18^c^	–2.51 ± 0.22^d^	13.33 ± 0.15^a^
b	−1.74 ± 0.83^b^	–13.43 ± 0.68^d^	3.71 ± 1.51^a^	–6.52 ± 1.09^c^

Values are the means ± standard deviation of triplicate determinations. Means with the different letters (a, b, c, or d) in the same row are significantly different at p < 0.05. NFT, novel fermented tea; BGT, Boseong green tea; TOT, Tieguanyin oolong tea; LBT, Lipton black tea.

**Table 7:** Sensory evaluation of different tea samples.

Tea	Color	Aroma	Taste			Overall Acceptance
			Sweetness	Astringency	Bitterness	
NFT	4.50 ± 0.66^a^	3.12 ± 0.93^b^	2.38 ± 1.06^a^	2.92 ± 1.21^b^	2.25 ± 1.11^b^	2.96 ± 1.00^a^
BGT	3.13 ± 1.23^b^	3.79 ± 0.83^b^	1.63 ± 0.88^b^	3.92 ± 1.02^a^	3.63 ± 1.21^a^	2.96 ± 0.91^a^
TOT	3.42 ± 0.72^b^	3.17 ± 0.92^b^	2.46 ± 1.22^a^	2.96 ± 1.20^b^	2.42 ± 1.06^b^	3.13 ± 0.85^a^
LBT	4.25 ± 0.61^a^	4.50 ± 0.59^a^	2.63 ± 1.17^a^	2.58 ± 1.18^b^	2.29 ± 1.12^b^	3.33 ± 1.05^a^

Values are the means ± standard deviation of triplicate determinations. Means with the different letters (a, b, c, or d) in the same column are significantly different at p < 0.05. NFT, novel fermented tea; BGT, Boseong green tea; TOT, Tieguanyin oolong tea; LBT, Lipton black tea.
